# Adult attention-deficit/hyperactivity disorder and nicotine use: a qualitative study of patient perceptions

**DOI:** 10.1186/1471-244X-14-141

**Published:** 2014-05-16

**Authors:** Michael Liebrenz, Anja Frei, Carl Erik Fisher, Alex Gamma, Anna Buadze, Dominique Eich

**Affiliations:** 1 Department of Psychiatry, New York State Psychiatric Institute, Columbia University Medical Center, 1051 Riverside Drive, New York, NY 10032, USA; 2 Psychiatric University Hospital, Division of ADHD Research, Lenggstrasse 31, 8032 Zurich, Switzerland; 3 Institute for General Practice and Health Services Research, University of Zurich, Pestalozzistrasse 24, 8091 Zurich, Switzerland

**Keywords:** Adult ADHD, Nicotine use, Explanatory models, Reasons for use, Qualitative

## Abstract

**Background:**

Adult Attention-Deficit/Hyperactivity Disorder (ADHD) is associated with high rates of comorbid substance use disorders, and cigarette smoking has a particularly high prevalence in this population. However, there is an ongoing debate as to whether this tobacco use is an attempt at “self-medication” or due to behavioral disinhibition. There is a surprising lack of qualitative studies that investigate the subjective perceptions of adults with ADHD regarding cigarette smoking. The present study was designed to fill this gap in the literature.

**Methods:**

We recruited twelve adult patients with ADHD and comorbid tobacco use from our ADHD consultation service, an outpatient facility of the Zurich University Psychiatric Hospital. Subjects were interviewed using qualitative methodology, and Mayring's qualitative content analysis was used to evaluate findings.

**Results:**

We identified two explanatory models linking ADHD and tobacco use: *smoking as an attempt at self-medication and “smoking as a social behavior”.* On one hand, subjects considered tobacco a therapeutic aid, reporting positive effects on “inner tension” and cognitive function, and noted possible antidepressant properties as well. On the other hand, subjects considered smoking to enhance social functioning and to have a positive impact on interpersonal relationships. The majority believed that stimulant medications offered only a transient decrease in patterns of tobacco use because their ability to reduce nicotine cravings wore off quickly. Others believed that stimulants had no effect or even reinforced cigarette use.

**Conclusions:**

Participants had different views about the link between cigarette smoking and ADHD. While the majority thought of nicotine as a sort of therapy, viewing smoking as a way to self-medicate symptoms of ADHD, motivations for nicotine use were also related to self-image, desire to belong to a peer-group, and a drive to undermine perceived social norms. Ultimately, these findings can be used by clinicians to improve treatment alliance and collaboration.

## Background

It is well established that Attention-Deficit/Hyperactivity Disorder (ADHD), a highly prevalent neuropsychiatric disorder that begins during childhood, largely persists into adolescence and adulthood [1-3]. ADHD is characterized by a diverse range of psychosocial impairments [4] and is highly comorbid with a wide range of other mental disorders. The most prevalent of these are mood disorders, anxiety disorders, impulse control disorders, and substance-use disorders (SUD) [5-7]. In adults with persistent ADHD, the prevalence of a comorbid SUD has been estimated at 47% or even higher in some series [8-10]. Furthermore, patients with ADHD show significantly higher rates of cigarette smoking than do members of the general population (35 - 55%) [11-13], as compared to 19% - 40% [14-16].

A larger epidemiological study was conducted to obtain knowledge about the association between ADHD and tobacco consumption in a Swiss sample of adult ADHD patients; previously, research on this subject had stemmed primarily from North America. Our findings were based upon complete data from 100 adult ADHD patients. In this study, which is only published in German, we reported a significantly elevated rate of current smokers in our sample (55%), as compared to 31% in the general Swiss population [13].

There is ongoing debate in the research community whether this ADHD-associated tobacco use is an attempt at “self-medication” (i.e., to attenuate symptoms of inattentiveness and improve executive function and cognitive performance), if it is simply a consequence of an underlying deficit in the ability to inhibit maladaptive impulses [17], or if the elevated risk for SUD (in general) is a “discrete dimension” [18] of inattention [19] or impulsivity [20]. Moreover, there are contradicting reports on the effects of stimulant medications on smoking behavior among adults with ADHD. Some reports point toward no effect [21], or a very modest decrease in tobacco consumption [22], while other authors associate stimulant treatment with increased tobacco use and nicotine craving in healthy volunteers [23], as well as in affected individuals [24].

The findings of some studies support the self-medication argument that nicotine improves self-rated vigor and concentration as well as performance on objective tasks, including chronometric measures of attention and timing accuracy [25-27]. Furthermore, deficits in sustained attention are among the most consistent findings in studies of the cognitive deficits associated with ADHD [28]; considering that nicotine has positive effects on sustained attention, some authors have argued that patients with ADHD use cigarettes to ameliorate a deficit in this function [29]. Aside from nicotine’s generally positive effect on cognitive function [30], smoking has also been linked with self-medication of emotional dysfunction in ADHD [31].

As to the behavioral disinhibition argument, some investigators report that ADHD is a specific, independent risk factor for tobacco use in the clinical samples they studied, after controlling for comorbid conduct disorder (CD) [10,32]. However, other authors suggest that orbitofrontal dysfunction and disinhibition are associated with antisocial behavior and related personality traits, and therefore with tobacco use [33,34]. Sousa *et al*. investigated a sample of 422 patients with adult ADHD and concluded that smoking initiation among patients with ADHD is associated with behavioral disinhibition beyond self-medication [17]. They also found that smoking on the part of these subjects was consistently linked to externalizing comorbid disorders such as CD and antisocial personality disorder.

In addition, Ivanov *et al*. suggest that the observed relationships among ADHD, CD, and SUD might result from the *impulsivity* present within each disorder, and concluded that underlying deficits in inhibitory control might play a central role in many of the behaviors associated with a high risk for SUD [18].

Supporting evidence for the self-medication and the disinhibition arguments has primarily been generated by means of quantitative research methods, such as epidemiological studies [11,32], systematic reviews [10], or clinical pharmacological trials [27,35]. Since studies of patients’ subjective perceptions have made valuable contributions to our understanding of other clinical issues, such as their perspectives on medication adherence and the causes of mental illness [36-38], the lack of qualitative research on the link between adult ADHD and cigarette smoking is surprising.

Smokers in the general population attribute their smoking to subjectively beneficial psychological and physiological effects, and they smoke more when they are in stressful life situations, are angry and anxious, or are depressed [39-41]. Furthermore, it is likely that tobacco use is heavily influenced by cultural factors such as race, acculturation, or socioeconomic status, beyond the pharmacology of nicotine, and frequently occurs as a consequence of a cluster of social behaviors that facilitate social interaction [42]. For example a recent study among a large social network of 12 067 people found that “smoking behavior spreads through close and distant social ties” [43]. It has also been widely reported that peer influences on smoking behavior are stronger among white adolescents than among other subgroups such as African American, Asian or Hispanic adolescents [44].

The current study explored how patients with adult ADHD, who currently smoked, viewed the relationship (− or link) between nicotine use and ADHD, using an inductive qualitative approach that made no initial assumptions about the relationship between ADHD and nicotine use. Thus, this study was not designed to test whether the above-described hypotheses regarding this link, identified using quantitate research methods, are consistent, but to “allow the research findings to emerge from the frequent, dominant, or significant themes inherent in raw data” [45]. We further explored how patients perceived the influence of prescription medications (both stimulants and non-stimulants) on patterns of tobacco use.

There is ample reason to believe that such qualitative investigations would be fruitful, especially in an effort to undertake a “collaborative or relationship centered treatment approach”, that allows for treatment providers and patients to allow a “mutual exchange of views” in an effort to solve problems in the patient’s best interest [46,47].

## Methods

### Sampling and recruitment

We recruited 12 participants from a larger epidemiological study of 134 adult patients with ADHD who had presented to the ADHD consultation service at the Centre for Addiction Disorders, an outpatient facility of the Zurich University Hospital, Switzerland [13,48].

In order to more thoroughly examine patients’ beliefs and perceptions about links between ADHD and cigarette smoking, we conducted a series of qualitative interviews using a purposeful sampling plan. All participants included in this study were adults with a diagnosis of ADHD and a current use of tobacco. They were also at least 18 years old and willing to give written informed consent for the study and the digitally recorded interviews. The sample was selected to provide diversity in relation to: (1) level of nicotine dependence (very low to very high); (2) clinical experience (previous in- and outpatient treatment episodes), including comorbidity (ICD-10 F3, F4 + F6); (3) gender (m/f) and age (25–52); (4) marital status (married, single, divorced); and (5) social class (professional, skilled, unskilled, unemployed, recipient of welfare or disability compensation). We also sampled for participants who had participated in a smoking cessation program (8) and for those who had not (4). Fifty-five participants of the larger epidemiological study qualified for inclusion. We were able to reach 48 of them and 12 agreed to participate.

Obstacles to study participation were rarely addressed by potential participants. Most often participants reported of a lack of time. In three cases, potential participants agreed to be interviewed, but failed to keep their appointment and could not be reached afterwards. Other potential barriers could have included a lack of compensation [49], a lack of interest in the specific research topic or a perceived lack of anonymity because of digital recording.

### Assessment of ADHD symptomatology

The diagnosis of ADHD was evaluated based on Utah criteria for diagnostic assessment, using the Wender Reimherr Interview (WRI) [50], translated into and validated for the German language by Rösler *et al*. and Retz-Junginger *et al*. [51-53]. Patients also received German versions of the Symptom Check List 90-Revised (SCL-90-R) [54], the Wender Utah Rating Scale (WURS-k) [52], and the Attention Deficit-/Hyperactivity Self-Report Scale (ADHS-SB) [55].

### Assessment of tobacco and other substance use

For each participant, the clinic’s complete chart was available, including biographical and psychiatric history, diagnoses according to the 10th revision of the International Classification of Diseases (ICD-10), and a detailed history of recent and lifetime substance-use patterns. Nicotine dependence among participants was further assessed with the 6-item Fagerstrom Test for Nicotine Dependence (FTND) [56].

### Qualitative interview

Participants were contacted by telephone to discuss the purpose of the study, obtain their informed consent, and arrange an initial interview. To allow for an atmosphere in which the participants felt free to fully express themselves, the interviews were then conducted at a location chosen by the participant [57].

We conducted single, semi-structured, in-depth interviews that lasted from 20–40 minutes, with an average duration of 30 minutes. Interviews began with narrative opening questions. A topic guide provided a flexible interview framework to explore beliefs that were not spontaneously covered in participants’ initial narrative. The guide addressed tobacco use patterns, reasons for tobacco use, influence of prescribed drugs on tobacco use, and the role and use of additional psychotropic substances. In addition, we allowed themes and motives identified during the first interviews of this qualitative study to be explored in the ones that followed, combining the principles of maximum variation and complexity reduction in order to simultaneously widen the scope of results and examine previous assumptions [58].

All interviews were conducted by the same researcher in Swiss German (an Alemannic dialect spoken in the “German-speaking” parts of Switzerland). They were digitally recorded and transcribed verbatim into Standard-German, since Swiss German is not a “written language” by AF. Transcripts were compared with recordings by the research team and validated with patients if necessary. Content analysis was carried out in German. Interpretation of findings and translation of selected quotes from German to English was carried out by ML. Translation errors (grammatical) were discussed between ML and CF, and corrected by CF. Subjects did not receive compensation for their participation.

All researchers had received training either as psychologists (AF) or as psychiatrists (ML, CF, AB, DE) and had previous research experience with qualitative methods.

### Analysis

Mayring's qualitative content analysis approach was used to evaluate findings. This framework constitutes a controlled approach for empirical and methodological qualitative analysis of texts within their context of communication, following content analytical rules and step-by-step models, without rash quantification [59]. In other words, we allowed the data to "speak for themselves," as opposed to approaching it with existing presumptions. Interview data were coded using an inductive qualitative procedure [60]. The resulting categories were discussed by the research team to validate ratings and achieve consensus. AF applied the final code, and consistency was confirmed through blind dual coding of transcripts with ML. If there was disagreement, researchers met to discuss and reconcile the coding. This did not become necessary until the late stages of revising the submitted manuscript and can be traced using the pre-publication history of this article on biomedcentral.com. Participant recruitment continued until we reached saturation of the data—i.e., there were no new themes emerging and we had tested all the categories for disconfirming variations. MAXqda software was used for text management and interpretation [61]. The study was authorized by the ethics committee of the canton of Zurich and all participants provided their written informed consent for it and the recorded interviews.

The topic guide is presented in Table 1.

**Table 1:** Topic guide

*Main questions*	“Can you tell me about your smoking?”
	“Have you ever thought about your reasons for smoking?”
	“What is the purpose of smoking?”
	“What are the effects if you smoke?”
	“In your opinion, is there a relationship between symptoms of ADHD and your personal patterns of smoking?”
	“If you used prescribed drugs for treatment of ADHD (and/or other mental disorders) now or in the past, did you notice a relationship between your use of these drugs and your patterns of smoking?”
*Additional questions*	“Did you (do you) notice any changes in (your symptoms of ADHD) when you were smoking?”
	“If you ever stopped smoking, did it have an effect on you? What kind? For how long?”
*Clarifying questions*	“Can you expand a little on this?”
	“Can you tell me anything else?”
	“Can you give me some examples?”

## Results

Participant characteristics, diagnosis, and tobacco consumption patterns are described in Table 2.

**Table 2:** Participant characteristics, diagnosis, and tobacco consumption patterns (n = 12)

**Name**^**a**^	**SC**^**b**^	**Age**	**Cigarettes per day at time of interview**	**Nicotine dependence**^**c**^	**ADHD subtype**	**ICD 10 comorbidity**	**Use of other substances**	**Current medication**	**Previous phases of nicotine abstinence**
Mr. A.	Yes	53	2 - 3	Very low	Combined	-	-	Stimulants	1 month (1993)
Mr. B.	Yes	42	3 – 5/week	Very low	Inattentive	F33.4	-	Stimulants, antidepressants	7 years (starting 1996)
Mrs. C.	Yes	25	20	Low	Combined	F12.24	Cannabis	Stimulants, antidepressants	15 months (2002)
Mrs. D.	Yes	46	20	High	Combined	F10.25	Alcohol	Stimulants, antidepressants	6 months (1993
						F11.20			
						F14.20			
Mrs. E.	Yes	27	20	Very high	Combined	-	-	Stimulants	4 – 5 years (2000)
Mrs. F.	Yes	47	23	Very high	Combined	F43.21	-	Stimulants, antidepressants	3 weeks (1994)
Mrs. G.	Yes	42	30	Moderate	Combined	F43.21	-	Antidepressants	One year (2001)
Mrs. H.	No	26	35	High	Combined	F43.21	-	Stimulants, antidepressants	18 months (2003)
Mr. I.	No	47	35	High	Hyperactive/impulsive	F12.24	Cannabis	Stimulants	1 month (2003)
						F60.2			
Mrs. J.	Yes	44	30	Very high	Combined	F11.202	Alcohol	Stimulants	6 months (1984)
						F41.2			
Mr. K.	No	46	33	Very high	Combined	F10.24	Alcohol	Stimulants	1 month (1992)
						F33.4			
Mr. L.	No	32	35	Very high	Combined	F10.25	Alcohol	Stimulants	None
						F14.20			

^a^pseudonym.

^b^participated in smoking cessation treatment.

^c^nicotine dependence according to Fagerström.

Of the 12 participants, seven were female and five were male. Their average age was 40, and they ranged from 25–53. At the time of the interview, all participants were currently smoking cigarettes, but their patterns of smoking varied greatly (from a minimum of 3–5 a week to a maximum of 35 a day), as did the severity of their nicotine dependence, according to the FTND (from very low to very high).

Ten participants had the combined type of ADHD, one had the predominantly inattentive type, and one had the predominantly hyperactive-impulsive type. All but two had another comorbid mental disorder. The most common comorbidities were SUD (other than nicotine dependence) and affective disorders. Six participants (50%) were employed, two (16%) were students, and four (33%) were unemployed or had an uncertain employment status.

In our analysis of the interview data, we identified two main themes linking ADHD and tobacco use: *smoking as an attempt at self-medication, and smoking as sensationalism, the search for a positive self-image and peer-group-mediated behavior.* Examples of these themes follow, but it bears noting that there was significant overlap among themes: some participants identified more than one specific link between ADHD and smoking and had adopted a multifaceted explanatory model to describe the relationship. Following the description of those themes, we also describe participants’ beliefs about the influence of prescription drugs and about their experiences with other psychotropic substances.

### Overall beliefs about the link between ADHD and tobacco use

The majority of participants readily acknowledged that cigarette smoking had psychological and physiological effects on them. Nine study subjects described a link between ADHD and tobacco use, but one participant reported that he had not thought about a connection:

“I don’t know, and I don’t want to lie to you. Maybe, I really don’t know. I cannot really judge it, because I have always been the same, not one time with ADHD and one time without it.”

Mrs. G.

Two participants did not address this subject in their narratives. In order to avoid leading questions and to preserve a non-judgmental stance, participants were not pressed on this subject.

### Theme I: Smoking as an attempt at self-medication

Most frequently, subjects acknowledged a link between ADHD and tobacco use by giving reasons for smoking cigarettes. Upon further exploration of the effects of nicotine consumption, participants expressed very different but generally positive views of those effects. Many attributed their cigarette use to general feelings of stress or being overwhelmed in different social contexts:

“…If I am getting out of some sort of stressful situation and I get the feeling my brain ‘rotates,’ then I get the feeling that I should go have a cigarette and then one can see the world much clearer.”

Mr. K.

Other study subjects used cigarettes specifically to reduce inner tension, to treat symptoms of restlessness, and for relaxation purposes:

“It reduces tension, I even believe partially. It really depends on the moment, but I would even say it relaxes the muscles. Especially in those moments when you have not smoked for a while and you absolutely want to smoke, then you can notice this.”

Mrs. C.

Participants repeatedly commented on the positive effects they perceived in relation to their ability to concentrate, be attentive, and solve problems. Two subjects also compared nicotine’s effects to those of other substances.

“…and you know earlier I was using other [illegal] drugs, and you know every time I had to do something, on which I absolutely had to concentrate on, something minor, then I would take heroin. Then I could behave and concentrate. And it could have been the most boring stuff in the world, for example. And you know a cigarette has a very similar effect, like alcohol, like some sort of sedative, which has the effect on me that I can do something really boring, something where I have to crunch numbers for hours at a time, then [cigarettes] help me with that.”

Mrs. J.

One participant with major depressive disorder attributed mood-stabilizing properties to nicotine:

“And you know, I am not accurately diagnosed with ADHD. I am somewhere in between a depression and ADHD, so this is an area where no one can say exactly, and you know, I think that I come more from the depressive side, in that sense it is a form of self-medication. It is a surrogate to comfort yourself and to retreat. Alone with one’s cigarette – it is almost like you can solve all the problems of the world, somehow, I think.”

*Mr. B*.

Another study subject associated a difficult upbringing, including emotional neglect by his parents, with cigarette smoking; he felt that it took the form of self-medication for emotional dysfunction.

“I always had the feeling that I got too little emotional warmth. Maybe it is because my mother was overwhelmed, because I was an ADD child…but something was missing all my life, despite the fact that I have an intact marriage, children, family, and social stability. I am compensating for something that is missing. And if I can make that happen with 2–3 cigarettes, then I am very happy with that.”

Mr. A.

### Theme II: Smoking as a social behavior

Subjects also frequently expressed the view that smoking had positive effects on interpersonal relationships, a valuable asset for socializing that could be used as a way of connecting to others:

“…smoking gives me a feeling of belonging and togetherness, something I can really enjoy, so I can lay back and smoke one [cigarette]…I find it very pleasant to be together with a group of people and everybody says, let’s go, we will have a smoke, then I like it.”

Mrs. G.

Some participants had a completely different view, primarily associating their tobacco use not with a search for specific effects but with a desire to take risks, to try something new with the appeal of the forbidden. We label this subtheme “sensationalism.” One study subject believed that patients suffering from ADHD are more likely to use psychotropic substances in general. (Of note, this subtheme has some overlap with views and perceptions regarding the initiation of smoking and its effects on interpersonal relationships.)

“Generally speaking, if I compare myself to others, I am less fearful. My readiness to assume risk is just higher compared to others, which is also a small part of the reason I began to smoke. Although you know that everything is harmful and so on, but the appeal of the forbidden, to begin with, is something symptomatic for individuals with ADHD, this wanting to know how it really is, this experimenting and this behavior…”

Mr. B.

This subtheme is further illustrated by two female participants, who described themselves as “rebellious” and “revolutionary,” and expressed a desire to subvert perceived social norms. Furthermore, they associated smoking with a positive self-image.

“…it might be connected to the fact that I always was a bit of a misfit as child, and later more into revolution and rebellion. And as a smoker you were somehow always more on the unhealthy side, but this was very clear to me, and for years I thought ‘I don’t want to quit smoking, the earlier I die…’ and so on…[started giggling], yes, and I think it has to do with the fact that the people who smoke are not the usual ones…and I identified myself with that…and I never had a self-perception as a non-smoker…that did not fit…”

Mrs. C.

Unlike the participants who were looking for a sense of belonging, other study subjects who had initiated their tobacco use in adolescence voiced explanations that emphasized their search for a self-image of “coolness” among a group of peers, even if they found smoking repelling.

“It was a process. Initially I found it disgusting, but I wanted to belong to that group of people… I really did have the feeling then, with smoking, that I am one of the more cool people with a more laid-back style.”

Mrs. G.

### Influence of prescription drugs on tobacco use patterns

All participants had some experience with prescribed psychotropic medications, mostly stimulants and antidepressants. We describe these findings separately but do not label them as a theme, because they do not present explanatory models linking ADHD and tobacco use. Subjects generally believed that medications had affected their tobacco use patterns, though the medications’ influences were experienced and expressed quite differently. Some participants reported that stimulant therapy (e.g., methylphenidate) initially reduced their tobacco use patterns:

“… in the very beginning, right when I started, I had the feeling that it did [decrease the desire for smoking], but this effect wore off quickly. Yes, the first day I had like no desire. Maybe I should take more Ritalin [methylphenidate].”

Mrs. D.

Other subjects believed methylphenidate increased their craving for smoking cigarettes:

“… I actually had the feeling that I was smoking more cigarettes when I started Ritalin. Although I don’t know how closely this is connected, but I did have the feeling that it is, because I felt I received for a time too high of a dosage, and then when I had little highs, I noticed I smoked or drank more.”

Mrs. H.

A minority did not notice any difference:

“… No my smoking patterns did not change much when I started Ritalin, I am like that, it is a habit, it is not about the smoking really. It is more, I answer the phone and I have a cigarette, or I sit in front of my computer and then I have one in my hand, I am not even smoking it, because I am typing. Then my keyboard is full of ashes, and smoke is in my nose but not because I am inhaling, but just because the cigarette is around…”

Mrs. J.

One study subject experienced a medication with stimulant-like properties as so calming that her desire for cigarette smoking abated:

“…I have the impression that, since Dexamin [dexamphetamine], calms me down, calms me down very much. I have the feeling that smoking became less important and played only a minor role, because for a time I was not smoking excessively. Yes, because I had the feeling that I was calm, that smoking was not that important anymore. I was still smoking out of habit, but it still had an influence. Since with Ritalin I was partially still very nervous and then smoking was there, this goes hand in hand…”

Mrs. E.

Another participant had this experience with the use of an antidepressant:

“… Really, I did not notice an effect at all and I had been trying with Dr. Eich [DE] for three years now. And the first time that I had the feeling that [medication] was useful was with Fluctine [fluoxetine], that gave me more of a balance…until now I had to say, it is not working for me, Concerta [methylphenidate] did not at all…”

Mrs. G.

## Discussion

In this qualitative study, nine out of twelve subjects clearly identified perceived links between tobacco use and their ADHD. One had not thought about a connection and two participants did not address this topic in their narrative. In addition, subjects described an influence of prescription medications, as well as an effect of other psychotropic substances on their thoughts and behaviors related to tobacco use.

We identified two core beliefs linking ADHD and cigarette smoking. The first theme, *smoking as an attempt at self-medication*, was adopted by the majority of the participants as their preferred description of the link between smoking and ADHD. Subjects generally had a positive view of the effects of tobacco, describing a range of beneficial uses: reducing tension, alleviating restlessness, relaxing in general, improving attention, regulating emotional distress, and relieving depressive symptoms. Thus participants might use tobacco to try to treat the symptoms and cognitive deficits arising from adult ADHD [62,63]. These qualitative reports are in accord with previous findings that nicotine may improve clinical symptoms and cognitive function in adults with ADHD [26,64]. Overall, our results provide further support for the hypothesis that smoking is a form of self-medication among adults with ADHD [65].

The second theme, *smoking as a social behavior* demonstrated that smoking was considered by many to enhance social functioning and to have a positive impact on interpersonal relationships.

Furthermore, some participants primarily initiated tobacco use not to attenuate symptoms of inattention or hyperactivity but to live a more exciting lifestyle, to undermine perceived social norms, to enhance their self-image, and to gain access to a desired non-conformist peer group. It is possible that the ADHD participants in this study who favored this motive are more impulsive and behaviorally disinhibited than others [17]; however, none fulfilled ICD-10 criteria for a conduct disorder in the past, or a current personality disorder. It must be noted that subjects who identified this motive began smoking against the backdrop of their overall subjective experiences, cultural norms and institutions, gender roles, and aesthetics, all of which are strong influences on smoking behavior in general [42,66].

At first glance, the views of our study participants do not differ greatly from explanations given by cigarette-smokers without ADHD. For example, a recent qualitative study of cigarette-smoking college students found that smoking “served as an aid in alleviating anticipated stress”; “helped clear the mind when shifting from one subject to another”; “helped to refocus thoughts during a study session, facilitating greater concentration”; “served as a reward to celebrate the completion of a study session or an examination”; and helped to change the mindset when “transitioning from studying to being social” [67]. However, it must be noted that nicotine effects in adults with ADHD might exceed those in healthy volunteers, because they improve the attention of the former, as well as their clinical symptomatology [68]. Comparisons with healthy volunteers or the general population might therefore be misleading.

Finally, findings on the *influence of prescription drugs on tobacco use patterns* were heterogeneous. Given that existing literature on the effects of stimulant medication on smokers with ADHD presents conflicting conclusions, we were intrigued to learn how subjects would describe the influence of stimulant medication on their tobacco-use patterns. However, only a few subjects made a clear assertion. The majority believed that the effects of stimulant therapy on nicotine craving wore off quickly, resulting in only a transient decrease in smoking, or that stimulants had no effect on cigarette use or even reinforced it. This supports Hurt et al., Winhusen et al., and Rush et al. [21-23].

Furthermore, Vansickel *et al*. recently reported that immediate-release methylphenidate used by smokers with ADHD actually increased both the total number of cigarettes smoked and their carbon monoxide levels [69]. Other investigators did not find significantly increased daily smoking rates in adults with ADHD [22]. It has been suggested that differences in formulation might explain this discrepancy [22]. In our sample, we did not find evidence for a link between smoking patterns and stimulant formulations.

We acknowledge the limitations of this study. First, we wished to obtain a comprehensive understanding of participants’ views, so we conducted extensive interviews with a small sample. Second, all study subjects were recruited from the same outpatient treatment facility, so it is unclear to what extent the present findings can be generalized. Third, since a majority of potential participants could not be interviewed for this study, there may have been an additional self-selection or non-response bias, further limiting generalizability. Fourth, interviews were conducted by a clinical psychologist and analyzed by a team of psychiatrists and psychologists actively involved in multimodal treatment for patients with ADHD, which may have influenced categorization.

The results we have presented should therefore be verified by further studies with more diverse patient groups. An initial step could be to recruit participants from more diverse treatment modalities, younger age group (e.g. adolescents 15 – 18 years) and with a wider variety of comorbidities. Since the majority of our participants belonged to the adult ADHD combined subtype, further research could also focus on the inattentive and hyperactive subtypes or presentations according to DSM-V. Finally, we did not include a non-ADHD comparison group, which could significantly enhance our understanding of the perceived differences in nicotine effects between adults with ADHD and the general population.

## Conclusion

Adults with ADHD and comorbid tobacco use expressed different views on the link between cigarette smoking and their ADHD. While the majority believed that nicotine has a positive effect and considered smoking an attempt at self-medication of ADHD symptoms, nicotine use was also linked to a desire for “sensationalism”: the search for a positive self-image, desire to belong to a peer-group, and a wish to undermine perceived social norms. Uncertainty and diverse opinions arose in relation to the effect of stimulant medications on patterns of tobacco use. Some subjects believed that stimulants were helpful for smoking cessation but that their positive effects wore off quickly, while others felt that stimulants either had no effect or worsened smoking.

Taken together, our findings suggest that neither the self-medication hypothesis nor the behavioral disinhibition model alone completely explains the link between ADHD and cigarette use. We found support for the self-medication hypothesis, but social and cultural factors were also highly influential. Studies of other mental disorders have established that patients prefer to draw on their own experiences, and that treatment approaches that do not take patients’ subjective perceptions into account are unlikely to increase help-seeking behavior [70]. We therefore suggest that clinicians who treat adult patients with ADHD and comorbid tobacco make use of the findings reported here to work for optimal treatment alliance and collaboration [71-73].

## Competing interests

We declare that we have no conflicts of interest.

## Authors’ contributions

DE and AF contributed to the design and the coordination of the study. All authors contributed to interpretation of data. ML prepared a first draft of the manuscript. All authors read and approved the final version of the manuscript.

## Pre-publication history

The pre-publication history for this paper can be accessed here:

http://www.biomedcentral.com/1471-244X/14/141/prepub
